# Conjugal amyotrophic lateral sclerosis: a case report from Scotland

**DOI:** 10.1186/s12883-017-0847-9

**Published:** 2017-03-29

**Authors:** P. M. Fernandes, M. R. Macleod, A. Bateman, S. Abrahams, S. Pal

**Affiliations:** 1 Centre for Clinical Brain Sciences, University of Edinburgh, Edinburgh, UK; 2 Centre for Clinical Brain Sciences, University of Edinburgh & NHS Forth Valley, Edinburgh, UK; 3 Department of Critical Care Medicine, Western General Hospital, Edinburgh, UK; 4 Psychology – School of Philosophy, Psychology and Language Sciences, Anne Rowling Regenerative Neurology Clinic, and Euan MacDonald Centre for Motor Neurone Disease Research, University of Edinburgh, Edinburgh, UK; 5 Centre for Clinical Brain Sciences, Anne Rowling Regenerative Neurology Clinic, and Euan MacDonald Centre for Motor Neurone Disease Research, University of Edinburgh & NHS Forth Valley, Edinburgh, UK

**Keywords:** Case report, Conjugal, Amyotrophic lateral sclerosis, Motor neuron disease

## Abstract

**Background:**

Conjugal amyotrophic lateral sclerosis is rare, with significant effects on psychological and care needs. We report a case of conjugal amyotrophic lateral sclerosis disease from central Scotland. This case is particularly unusual as both patients were diagnosed within an 18-month period and experienced the disease simultaneously, with similar symptomatology and progression.

**Case presentation:**

Patient A was a 71-year-old man who presented with unilateral arm weakness and wasting. Patient B was a 68-year-old woman who presented with unilateral shoulder and elbow weakness. Diagnosis of amyotrophic lateral sclerosis was made within a few months of presentation in both cases, based on typical clinical symptomatology together with supportive neurophysiological testing. Interventions included enteral feeding and non-invasive ventilation. The time period between symptom onset and death was 5 years for Patient A and 3.5 years for Patient B.

**Conclusion:**

This case illustrates two main points: the care issues surrounding cases of conjugal neurological disease, and the psychological issues in these patients.

There are significant care issues arising when co-habiting couples both develop severe functionally limiting neurological diseases at the same time. The more slowly progressive nature of Patient A’s disease may be at least partially explained by the support he was able to receive from Patient B before she developed symptoms. Secondly, there are important psychological effects of living with someone with the same – but more advanced – progressive and incurable neurological disease. Thus, Patient B was reluctant to have certain interventions that she had observed being given to her husband. Lastly, no plausible shared environmental risk factors were identified, implying that the co-occurrence of ALS in this couple was a random association.

## Background

Amyotrophic lateral sclerosis (ALS) is a progressive neuro-degenerative disease affecting motor neurons in the cortex, brainstem, and spinal cord, and is associated with fronto-temporal dementia. ALS is rare, with data from the Scottish Motor Neurone Disease Register [1] indicating an annual standardised incidence of 2.40 (95% C.I. 2.22-2.58)/10^5^. Cases of conjugal ALS are especially rare, with few published reports [2–8]. These cases pose unique psychological and care challenges, with opportunities to apply the lessons learnt from these situations to other areas of neurological care.

ALS can be divided into familial and sporadic variants, with the latter accounting for 90% of cases. Associated genes include SOD1, C9orf72, TARDBP, FUS, and OPTN [9]. The aetiology of sporadic ALS remains unclear; cases of conjugal ALS may therefore offer further insights into the aetiology of this condition, including the role of environmental factors.

We report a case of conjugal amyotrophic lateral sclerosis disease from central Scotland. This case is particularly unusual as both patients were diagnosed within an 18-month period and experienced the disease concurrently, with similar symptomatology.

## Case presentation

### Patient A

A 71-year old right-handed Scottish man presented in 2010 following a six-month history of unilateral arm weakness and wasting, with associated muscle cramps. His occupational history included the navy, a power plant, and delivery driving, but there was no history of exposure to specific chemicals. His father had similar symptoms before his death (from thrombosis) aged 72 but was never formally diagnosed with ALS. Examination showed asymmetrical weakness of elbow flexion and extension, with marked upper arm wasting but preserved reflexes and bulbar function.

Formal neuro-psychological testing showed preserved cognition (Edinburgh Cognitive Assessment Scale [ECAS] [10] score 118) with some evidence of emotional lability (Emotional Lability Questionnaire Score [ELQS] [11] 11). Hospital Anxiety and Depression Scale (HADS) [12] results showed evidence of clinical levels of anxiety (score 12/21) and borderline depression (score 9/21). Electrophysiological testing revealed widespread denervation consistent with ALS; other investigations were unremarkable, including genetic testing for expansions/mutations in genes commonly associated with ALS (including C9orf72, SOD1, TARDBP, FUS, and OPTN). The progression of his disease is described in Table 1; death occurred 5 years after symptom onset.

**Table 1:** Comparison of disease progression for Patient A and Patient B

	Patient A	Patient B
0 months	Onset of symptoms	
6 months	Presentation to neurology clinic	
10 months	Diagnosis of ALS	
15 months	Progression of arm symptoms	
	Bilateral leg weakness	
18 months	Bilateral flail arms	Onset of symptoms
	Postural dyspnoea	
22 months	Commenced nocturnal non-invasive ventilation (NIV)	
25 months	Commenced daytime NIV	Presentation to neurology clinic
	Swallowing difficulties	
	Unable to walk	
26 months	Insertion of radiological inserted gastrostomy tube	Diagnosis of ALS
30 months		Progression of symptoms
		Unable to dress or eat independently
53 months		Insertion of percutaneous endoscopic gastrostomy tube
59 months	Commenced 24-hr NIV	Commenced on intermittent NIV
		Bilateral flail arms
		Unable to walk more than a few steps
61 months	Death	
62 months		Death

A post-mortem confirmed ALS with spinal cord ventral root atrophy and widespread loss of anterior horn cell neurons, with remaining cells containing TDP-43 positive inclusions. No TDP-43 positive inclusions were seen elsewhere in the brain or spinal cord.

### Patient B

A 68-year old right-handed Scottish woman presented in 2012 following a nine-month history of progressive shoulder and elbow weakness. She was the wife, and main carer, of Patient A, and presented when her symptoms made caring for her husband difficult. There was no family history of ALS and she was not a blood relation of her husband. No specific environmental exposures were reported. Examination showed bilateral wasting and weakness of shoulder girdle musculature and deltoids, with diminished reflexes and widespread fasciculation. Neuro-psychological testing showed normal cognition (ECAS score 110), and no evidence of emotional lability (ELQS score 0) or anxiety or depression (HADS scores 7 and 6 respectively). Electrophysiological findings were consistent with ALS; other investigations were unremarkable, including genetic testing. Her disease progressed in a similar fashion to her husband, as shown in Table 1. Notably, Patient B was reluctant to have enteral feeding or ventilatory support after witnessing the impact of these interventions on her husband. Death occurred 3.5 years after symptom onset; a post-mortem was not performed.

## Discussion

This case illustrates three points: the care issues surrounding cases of conjugal neurological disease, the psychological issues in these patients, and the possibility of finding shared environmental risk factors.

Patients with ALS require increasing functional assistance over time, with family members often providing much of this care; the first indication of Patient B’s illness was progressive decline in her ability to support her husband. The impact of functional decline is synergistically amplified when both patients suffer from progressive neurological conditions, especially where deficits are mirrored as in this case. A major challenge for all involved was managing the lifestyle changes resulting from such a complex situation; this includes equipment acquisition and storage, and arrangement of two packages of personal care support. Fully co-ordinating care between both patients, including respite facilities and outpatient appointments, was helpful. Table 1 compares clinical progression for both patients, showing that Patient A had a longer disease course than Patient B. This may be due to underlying differences in disease tempo but may also reflect the additional support Patient A received from Patient B before she became symptomatic.

This case demonstrates the psychological issues arising when treating patients with the same progressive and incurable disease who live together, particularly when one patient’s condition is more advanced. Patient B was reluctant to commence non-invasive ventilation and enteral nutrition, having observed some of the negative implications of these on her husband (rapid progression to full ventilator dependence and technical difficulties with enteral feeding). Furthermore, caregivers perceive ALS interventions more negatively than patients [13]; her observations whilst caring for her husband will have affected her own future choices. Additionally, although neither patient showed cognitive changes, Patient A’s clinically significant levels of anxiety, emotional lability, and borderline depression may have affected the ability of his wife to support him. Interestingly, ALS patients have similar quality of life scores to their carers [13]. This may be because caregiving is associated with negative effects on carer health, including emotional stress and adverse psychological state [14]; caregiver burden increases with duration of caregiving [15]. Scant information is available on the psychological impact of conjugal terminal disease, especially regarding caregiver burden, presumably because of the rarity of these situations.

Conjugal cases of the same disease may offer insights into environmental risk factors, as has been shown in Parkinson’s disease [16]. The lifetime probability of conjugal ALS has been calculated as 1/510,000 couples [8], giving an approximate rate of 0.75 couples/year [17]; concurrent disease, as in our patients, will be still rarer. The Swedish Multi-Generation Register did not find an increased ALS risk in spouses of patients [18], suggesting effects of environmental factors are likely to be restricted to pre-marital life. In our patients no common environmental or genetic factors were found, implying that the co-occurrence of ALS in this couple is a random association. In conclusion, the rarity of simultaneous conjugal ALS means that much can be learnt from these cases, especially regarding the impact on the care and psychological needs of both patients.

## Abbreviations

ALS: Amyotrophic lateral sclerosis
ECAS: Edinburgh cognitive assessment scale
ELQS: Emotional lability questionnaire score
HADS: Hospital anxiety and depression scale
MND: Motor neuron disease
